# Development and validation of a screening tool for SPondyloArthritis Screening in Sub-Saharan Africa: SpASSS questionnaire

**DOI:** 10.1186/s12874-023-01966-w

**Published:** 2023-06-21

**Authors:** P Lebughe Litite, R. Westhovens, A. Nkodila, J. J. Malemba, K. de Vlam

**Affiliations:** 1 Rheumatology Unit, Department of Internal Medicine, Faculty of Medicine, University of Kinshasa, Kinshasa, Democratic Republic of the Congo; 2 Division of Rheumatology, University Hospitals Leuven, Louvain, Belgium; 3 Department of Public Health, Faculty of Family Medicine, Université Protestante Au Congo, Congo, Kinshasa, Democratic Republic of the Congo

**Keywords:** Spondylarthritis, Validation, Screening questionnaire, Sub-Saharan Africa

## Abstract

**Objective:**

To develop and validate a screening tool to identify patients with a high likelihood for Spondyloarthritis (SpA) in the Democratic Republic of the Congo (DR Congo).

**Methods:**

The development of the SpA Screening questionnaire in Sub Saharian Africa (SpASSS) questionnaire followed 3 steps: The item generation was carried out by a systematic literature review according to the PRISMA guidelines on the clinical manifestations of SpA, interviewing clinical experts and the classification criteria for Spondyloarthritis. The candidate questions were tested in a population of 50 consecutive patients with confirmed diagnosis of spondyloarthritis, in a control population of rheumatic disease excluding SpA and in a group of 200 non-rheumatic participants, randomly chosen in the general population for question reduction and validation. Descriptive statistical analyses were performed to assess socio-demographic characteristics and response distribution for each item. Their diagnostic performance was investigated using ROC curves. For validation, principal component analysis was performed using factor analysis. Referral strategy score for SpA was determined by adjusted Cronbach’s alpha coefficient.

**Results:**

Mean ± SD age of SpA cases was 41.8 ± 14.4 years, 56% were men compared to diseased controls 60.0 ± 12.5 years, 28.7% men (*p* < 0.001). 14/20 items showed a statistically significant difference (*p* < 0.05) between SpA cases and control groups. All items were factorable and 6 components were identified. Only the two first components (C1 with 8 items, C2 with 3 items) showed a significant threshold for reliability in detection of suspected SpA with a Cronbach's alpha of 0.830 and 0.708. All validated items of these two components showed the global reliability threshold with α-adjusted Cronbach calculated at 66.9%. The performance for correctly screening SpA was demonstrated with an area under the curve of 0.938 (0.884–0.991) and 0.794 (0.728–0.861) for C1 and C2 respectively.

**Conclusions:**

This validation and item reduction of the SpASSS questionnaire for SpA might identify patients to refer for case ascertainment and will help conducting future epidemiological and clinical studies in the DR Congo.

**Strengths and limitations of this study:**

• To the best of our knowledge, this is the first study in Sub-Saharan Africa based on local data to develop a screening tool for SpA in the population for epidemiological and clinical use.

• Referral strategies based on context-specific data are necessary to provide accurate case definition and epidemiological data, thus reducing methodological bias.

• In the SpA group, no discrimination was made regarding SpA subtypes, disease duration, activity and severity.

**Supplementary Information:**

The online version contains supplementary material available at 10.1186/s12874-023-01966-w.

## Highlights

- To the best of our knowledge, this is the first study in Sub-Saharan Africa based on local data to develop a SpASSS questionnaire as screening tool for SpA in the population for epidemiological and clinical use.
- Referral strategies based on context-specific data are necessary to provide accurate case definition and epidemiological data, thus reducing methodological bias.
- The SpASSS questionnaire screening is a validated referral instrument in Lingala for the detection of spondyloarthritis regardless the subtype of SpA.

**Supplementary Information:**

The online version contains supplementary material available at 10.1186/s12874-023-01966-w.

## Introduction

Spondyloarthritis (SpA) is a heterogeneous group of chronic inter-related inflammatory arthropathies affecting mainly the spine but also showing involvement of peripheral joints, entheses and extra-articular sites as eyes, skin and bowel [1]. Based on the developed *Assessment of SpondyloArthritis International Society* (ASAS) classification criteria, SpA can be divided into two subsets: axial SpA (axSpA) including ankylosing spondylitis (AS) and non-radiographical axial spondyloarthritis (nr-axSpA) and peripheral SpA including reactive arthritis (ReA), psoriatic arthritis (PsA), enteropathic arthritis and juvenile SpA [2]. Mainly AS and PsA are studied and a large variation in prevalence is reported [3-5]. These variations might be explained by demographical particularities but also by the use of different methodological characteristics such as the year of data collection, sampling frame and case finding used in the available studies [3, 8,6-]. In general, it is accepted that AS and PsA are rather scarce among African blacks due to the low prevalence of HLA –B27 [4, 5, 9]. The prevalence and incidence of other subtypes of SpA is poorly studied [3, 8, 9]. The lack of HLA-B27 in this particular population may influence disease course, i.e. less prevalent acute anterior uveitis, less radiographic progression and delay in diagnosis of HLA-B27 negative SpA as it is also the case in the Western world [10]. The AS patients currently from Africa are older at disease onset, lack extra-articular manifestations and many patients do not have a family history of AS [11, 12]. The potential different disease course in Sub-Saharan population in addition to lower availability of imaging facilities such as MRI may lead to lower sensitivity and specificity of the usual criteria to diagnose SpA in other parts of the world including the Sub-Saharan region. A completely different environment on top of a different genetic background [4, 13, 14] might be responsible for a different disease presentation. Until recently and certainly before the HIV pandemic, PsA was infrequently reported in sub-Saharan Africa [5, 15]. Since then the prevalence of PsA seems to increase due to a possible relationship between human immunodeficiency virus (HIV) infection and soft tissue rheumatic lesions as reported in HIV-positive Zambians [16]. Finally, the knowledge about SpA in the global medical community in Africa is still insufficient, possibly contributing to under-reporting the prevalence of SpA by the medical community.

A different organization of the health care systems in Africa and poor access for many people to care might additionally contribute to this underestimation of the prevalence [17]. To the best of our knowledge, no studies have been reported with valid data about the presence and characteristics of SpA in Sub-Saharan Africa at present. Quality data are needed for the detection and proper diagnosis of SpA in Sub-Saharan Africa and also specifically the Democratic Republic of the Congo (DR Congo), enabling a timely care and a reduction of the disease burden and related physical disability. As part of a broader scientific initiative in the DR Congo towards this group of diseases, the objective of this first study is the development and the validation of a screening tool for the identification of patients with a high likelihood of suffering from SpA. This screening tool will be further used in clinical settings and epidemiological studies in Kinshasa, DR Congo.

## Patients and methods

### Development of the SpA screening questionnaire

**Fig. 1:**
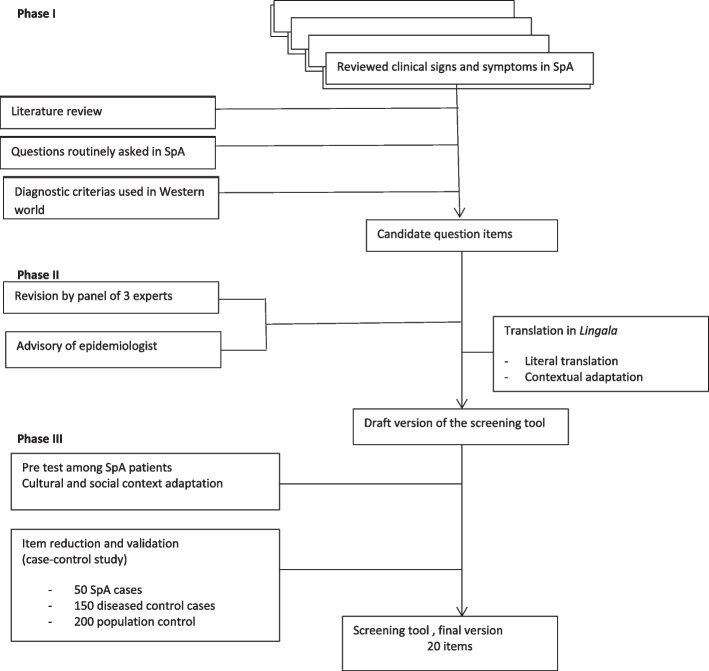
SpASSS questionnaire development overview

The screening tool development had 3 phases (Fig. 1). In phase I, we first reviewed clinical signs and symptoms seen among patients with SpA as determined by experienced rheumatologists. In DR Congo there is only one rheumatology unit located at the University Hospital of Kinshasa with 5 rheumatologists and assistants in training in rheumatology.

To start, potential question items were constructed via a systematic literature review combined with routinely asked questions in daily practice concerning SpA in DR Congo and based on the classification criteria of SpA in Caucasian subjects. A systematic literature review was conducted by PLL and JJM. A separate manuscript is prepared about the review process. Methods of the systematic review are briefly presented in a supplementary file 1 including the PRISMA diagram and AMSTAR2 evaluation [18]”.

In phase II, the face validity of the developed screening tool was reviewed and revised by a 3 experts’ panel in the field (RW, KDV and JJM). The modified question items were tested and revised if necessary. A notice of an epidemiologist was requested to estimate the validity of the items of this screening tool.

This validity consisted of comments regarding the flow of questions, typographical errors, wording/vocabulary, and meaning/comprehension for each item to ensure that they accurately described patients’ disease experience [5]. To assure the cross-cultural use, the items were translated in *Lingala*, the language spoken and understood by the general Congolese population. There were two essential steps: literal translation and contextual adaptation. The literal translation was done by 3 independent translators, having a different educational level and *Lingala* as native language. Then, the backward translation was performed into French by 3 other translators having French as native language using the three translated Lingala versions. This step led to a draft version of the screening tool that was consecutively evaluated in a pre-test phase among a small group of patients known to have SpA to adapt the different items to cultural and social contextual aspects in order to come to a final version of the screening tool. The SpASSS questionnaire is presented in supplementary file 2.

In phase III, a case–control study for validation and question item reduction was performed. The screening tool was piloted in subjects to check its validity in screening patients with different rheumatic diseases for its sensitivity and specificity for detecting SpA, with the clinical examination as the gold standard.

### Patients

Patients attending the University Hospital of Kinshasa (UHK) and the Provincial General Hospital of Kinshasa (PGHK) for rheumatic complaints were recruited during the period from March to April 2015.

Osteoarthritis was the most common rheumatic disease (62.2%), followed by soft tissue rheumatism (13.1%), gout (5.3%). The cumulative frequency of autoimmune diseases (rheumatoid arthritis, systemic lupus erythematosus, systemic sclerosis, dermatomyositis, and mixed connective tissues disease) and the frequency of osteoporosis were 4.2 and 3.7%, respectively. The frequency of SpA was 11.5%.

Fifty patients with a clinical diagnosis of SpA followed at the UHK and PGHK and aged 18 years or older. Two control populations were selected: 150 patients diagnosed with a rheumatic disease other than SpA were randomly recruited during their consultation follow-up and subsequently an additional group of 200 healthy participants randomly recruited from a district of the city of Kinshasa. Questions items were successively tested among patients with SpA, and in both control populations for validation and question item reduction. All patients consented to participate before inclusion. The study was conducted in accordance with the National Ethics Committee. The study received ethical approval from the National Ethics Committee (Comité national de bioéthique; ESP/CE/014/2016, Democratic Republic of Congo).

### Clinical assessment

All patients with SpA were assessed by a rheumatologist according to a standard protocol, including medical histories and physical examination. Details of joint symptoms, back pain and stiffness, personal and family histories of arthritis, skin lesions, symptoms of acute anterior uveitis, enthesitis, dactylitis, Crohn’s disease/colitis, diarrhea, urethritis, good reaction to non-steroidal anti-inflammatory drugs (NSAIDs) and a positive family history of SpA were recorded. All patients underwent conventional x-rays evaluation of the pelvis in anterior–posterior view. Sacroiliac joints were scored according to the modified New York criteria. X-rays of other joints were performed only if clinical involvement (arthritis, enthesitis, and dactylitis) was present. The Congolese version of the BASDAI and BASFI questionnaires were obtained by translating from French to Lingala by two rheumatologists. The translations reflected best the meaning of the French items. The measurements of the BASMI were made by the same rheumatologists.

### Statistical analysis

The socio-demographic characteristics and distribution of responses for each item were described as mean ± standard deviation for quantitative data and absolute and relative frequency for categorical data. The comparison of means of age and age at onset was performed using Student’s t-test. Pearson's Chi-square or Fischer's exact test were used to compare proportions of response distribution of different questionnaire items. The diagnostic performance of the items was investigated using the ROC curve with calculation of sensitivity, specificity, positive and negative predictive value. The *p*-value < 0.05 was the threshold of statistical significance. For questionnaire validation, principal component analysis was performed using factor analysis. The Keiser-Meyer-Olkin (KMO) index was used to measure the quality of the sample by indicating the quality of the correlations between the item questions. The Bartlett test of sphericity concluded that the item questions variables are globally dependent. The extraction coefficient was obtained from the analysis of variance of item questions. The determination of item components was performed using Varimax matrix rotation. The final validation of the items was done using the reliability and discriminant test with calculation of simple alpha Cronbach coefficients, convergent validity and discriminative validity values. Any component with an alpha Cronbach value < 0.70, convergent validity < 0.70 and discriminative validity < 0.70 was excluded from the questionnaire. The referral strategy score of SpA were determined using the adjusted alpha Cronbach coefficient. Statistical analyses were performed using SPSS for Windows version 22.

## Results

The analysis included a total of 400 participants: 50 SpA cases, 150 diseased controls suffering from other arthritis and a control population of 200 non-diseased participants (Table 1). The mean ± SD age of the SpA cases was 41.8 ± 14.4 years and 56% were men. The diseased controls were older (mean ± SD: 60.0 ± 12.5 years), and fewer were men (28.7%).

**Table 1:** Study population characteristics and distribution of answers of the screening tool among the different groups n (%)

Variables Items of questionnaire	SpA Cases *n* = 50	Non-SpA rheumatic controls *n* = 150	*P*	SpA Cases *n* = 50	Healthy population *n* = 200	*p*
Age	41.8 ± 14.4	60.0 ± 12.5	**< 0.001**	41.8 ± 14.4	33.1 ± 12.4	**0.010**
Age at onset disease	37.5 ± 12.5	55.7 ± 10.9	**< 0.001**	37.5 ± 12.5	41.4 ± 12.8	0.146
Sex						0.460
Male	28 (56.0)	43 (28.7)		28 (56.0)	116 (58.0)	
Female	22 (44.0)	107 (71.3)		22 (44.0)	84 (42.0)	
Do you have joint pain?			** < 0.001**			** < 0.001**
No	27(54.0)	16(10.7)		27(54.0)	172(86.0)	
Yes	23(46.0)	134(89.3)		23(46.0)	28(14.0)	
Do you have joint swelling?			**0.104**			** < 0.001**
No	27(54.0)	98(65.3)		27(54.0)	197(98.5)	
Yes	23(46.0)	52(34.7)		23(46.0)	3(1.5)	
Do you have joint swelling in more than 3 joints?			0.167			**0.026**
No	44(88.0)	121(80.7)		44(88.0)	193(96.5)	
Yes	6(12.0)	29(19.3)		6(12.0)	7(3.5)	
Are your legs affected ?			0.267			0.393
No	43(86.0)	121(80.7)		43(86.0)	166(83.0)	
Yes	7(14.0)	29(19.3)		7(14.0)	34(17.0)	
Do you have back pain?			** < 0.001**			** < 0.001**
No	8(16.0)	77(51.3)		8(16.0)	159(79.5)	
Yes	42(84.0)	73(48.7)		42(84.0)	41(20.5)	
Do you have stiffness in back lasting for > 30?			** < 0.001**			** < 0.001**
No	7(14.0)	143(95.3)		7(14.0)	199(99.5)	
Yes	43(86.0)	7(4.7)		43(86.0)	1(0.5)	
Do you have back pain a wakening you the 2^nd^ half of the night?			** < 0.001**			** < 0.001**
No	11(22.0)	147(98.0)		11(22.0)	192(96.0)	
Yes	39(78.0)	3(2.0)		39(78.0)	8(4.0)	
Does physical exercise improve your back pain?			** < 0.001**			** < 0.001**
No	12(24.0)	146(97.3)		12(24.0)	182(91.0)	
Yes	38(76.0)	4(2.7)		38(76.0)	18(9.0)	
Does NSAIDs improve your back pain?			** < 0.001**			** < 0.001**
No	7(14.0)	132(88.0)		7(14.0)	173(86.5)	
Yes	43(86.0)	18(12.0)		43(86.0)	27(13.5)	
Do you have anterior chest pain?			0.062			0.557
No	48(96.0)	150(100.0)		48(96.0)	190(95.0)	
Yes	2(4.0)	0(0.0)		2(4.0)	10(5.0)	
Do you have buttock pain?			** < 0.001**			** < 0.001**
No	21(42.0)	149(99.3)		21(42.0)	192(96.0)	
Yes	29(58.0)	1(0.7)		29(58.0)	8(4.0)	
Do you have red eyes now/in past?			** < 0.001**			**0.007**
No	36(72.0)	150(100.0)		36(72.0)	176(88.0)	
Yes	14(28.0)	0(0.0)		14(28.0)	24(12.0)	
Do you have heel pain?			** < 0.001**			** < 0.001**
No	30(60.0)	147(98.0)		30(60.0)	190(95.0)	
Yes	20(40.0)	3(2.0)		20(40.0)	10(5.0)	
Do you hav&e chronic diarrhea?			0.062			0.180
No	48(96.0)	150(100.0)		48(96.0)	198(99.0)	
Yes	2(4.0)	0(0.0)		2(4.0)	2(1.0)	
Do you have urethritis ?			** < 0.001**			** < 0.001**
No	41(82.0)	150(100.0)		41(82.0)	199(99.5)	
Yes	9(18.0)	0(0.0)		9(18.0)	1(0.5)	
Do you have psoriasis ?			0.250			0.200
No	49(98.0)	150(100.0)		49(98.0)	200(100.0)	
Yes	1(2.0)	0(0.0)		1(2.0)	0(0.0)	
Does one of your family members have AS?			-			0.800
No	50(100.0)	150(100.0)		50(100.0)	199(99.5)	
Yes	0(0.0)	0(0.0)		0(0.0)	1(0.5)	
Does one of your family members have psoriasis/uveitis/ chronic diarrhea?			-			0.800
No	50(100.0)	150(100.0)		50(100.0)	199(99.5)	
Yes	0(0.0)	0(0.0)		0(0.0)	1(0.5)	
Do you have nodules ?			0.750			0.407
No	50(100.0)	149(99.3)		50(100.0)	196(98.0)	
Yes	0(0.0)	1(0.7)		0(0.0)	4(2.0)	
Do you have dactylitis ?			**0.014**			**0.016**
No	46(92.0)	149(99.3)		46(92.0)	198(99.0)	
Yes	4(8.0)	1(0.7)		4(8.0)	2(1.0)	

Fourteen of the twenty items showed a statistically significant difference (*p* < 0.05) between the SpA cases and the 2 control groups. According to the results of the extraction values, all items were factorable with a KMO index and Bartlett's test of 0.718 and Chi-square = 1779.3 respectively (*p* < 0.001) (Supplementary Table 1).

Analysis of variance was used to extract the different coefficients for each item in the SpASSS questionnaire. The results showed that their coefficients were all greater than 0, they can thus be grouped into principal components according to the degree of association between each other.

The correlations between each of the validated item questions and the principal components derived from these correlations were estimated using the Varimax matrix rotation with identification of 6 principal components (from C1 to C6, Table 2). The first component explained 44.7% of the common variance, the others explained 36.0%; 5.6%, 5.0%, 4.4% and 4.3% respectively.

**Table 2:**
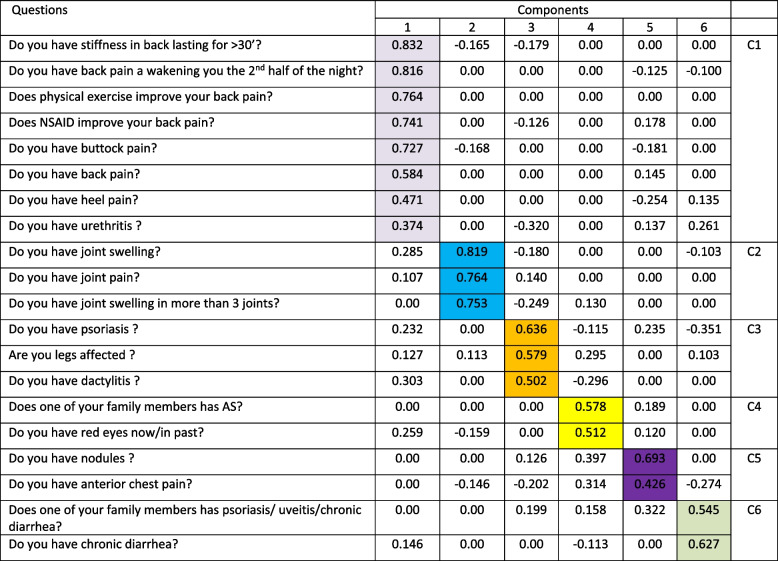
Matrix of components after rotation

After oblique rotation, the matrix of factor weights was a simple structure: the first component (C1) obtained important weights (> 0.360) with 8 items and weights close to zero with the other items. The second component (C2) obtained weights (> 0.750) with 3 items and low weights with the other items. The 3rd component (C3) had weights > 0.500 with 3 items; the 4th component (C4) had weights > 0.500 with 2 items, the 5th component (C5) had weights > 0.400 with 2 items and the 6th component (C6) had weights > 0.500 with 2 items and the other items had low weights close to 0. No item correlated with more than one factor. The interpretation of the factors shows that the items of the same component seem to share the same concept.

The final validation of the items was performed using the reliability and discriminant test with calculation of the coefficient of simple Cronbach’s alpha. Convergent and discriminative validity and reliability scale of each component items were reported in Table 3. Any component items with a Cronbach’s alpha value < 0.70 was excluded from the final validation questionnaire. Only components C1 (8 items) and C2 (3 items) showed a significant internal consistency threshold for reliability in the detection of SpA suspicious. After validation of these components, only the items of the first 2 components (C1 and C2) are validated with a Cronbach's alpha of 0.830 and 0.708 and success ranging from 88.3 to 89.5% for C1 and 83.3 to 88.1% for C2 (Table 3).

**Table 3:** Convergent and discriminative validity and reliability scale of each component items

Component	Number of items	mean ± SD	Α	Convergent validity	Discriminative validity
C1	8	0.98 ± 1.73	0.830	0.883	0.895
C2	3	0.76 ± 0.97	0.708	0.833	0.881
C3	3	0.39 ± 0.49	0.109	0.234	0.349
C4	2	0.19 ± 0.41	0.097	0.106	0.132
C5	2	0.18 ± 0.51	0.037	0.101	0.123
C6	2	0.15 ± 0.42	0.011	0.112	0.125

αCronbach’s alpha coefficient

mean ± SD value of reliability scale of each component items

**Table:** 

**C1: the 1** ^**st**^ **component** *Do you have stiffness in back lasting for* > *30 min?* *Do you have back pain awakening you the 2* ^*nd*^ *half of the night?* *Does physical exercise improve your back pain?* *Does NSAID improve your back pain?* *Do you have buttock pain?* *Do you have back pain?* *Do you have heel pain?* *Do you have urethritis ?*	**C2: the 2** ^**nd**^ **component** *Do you have joint swelling?* *Do you have joint pain?* *Do you have joint swelling in more than 3 joints?*
**C3: the 3** ^**rd**^ **component** *Do you have psoriasis?* *Are your legs affected?* *Do you have dactylitis?*	**C4: the 4** ^**th**^ **component** *Does one of your family members has AS?* *Do you have red eyes now/in past?*
**C5: the 5** ^**th**^ **component** *Do you have nodules?* *Do you have anterior chest pain?*	**C6: the 6** ^**th**^ **component** *Does one of your family members has psoriasis/ uveitis/chronic diarrhea?* *Do you have chronic diarrhea?*

The first two components thus validated show a higher rate of convergence, discrimination and reliability for the detection of suspected SpA.

Based on these two components, a new component regrouping all the validated items of these two components was developed and tested to deduce the global reliability threshold with α-adjusted Cronbach calculated at 66.9%. On 100% of the validated items retained for the screening tool of SpA, 66.9% internal reliability was found for a definite referral strategy model towards SpA (suspicious case).

The maximum score of the questionnaire was 11 corresponding to 100% of responses and the minimum score of 0 corresponding to no positive response. Taking into account the threshold of 66.9% of internal reliability defined by the α-adjusted Cronbach, the number of positive responses expected for referral strategy to suspect case of SpA should be ≥ 7 positive item responses.

**Fig. 2:**
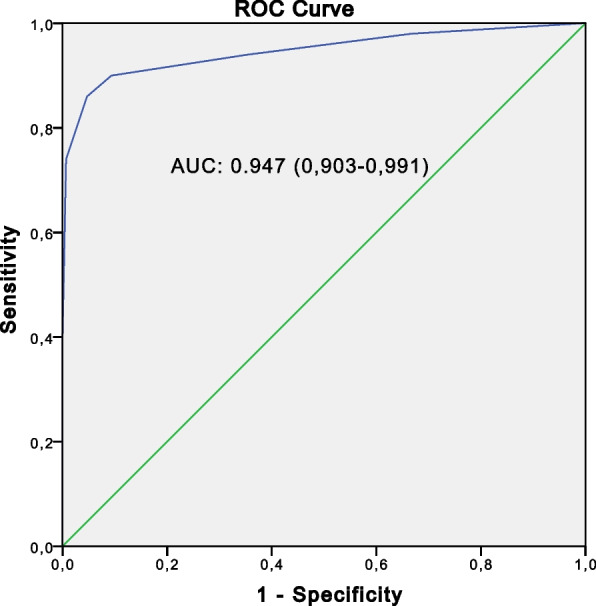
The receiver operating characteristic curve for the global component (C1 + C2) of SpA cases (*n* = 50) versus non-SpA rheumatic controls (*n* = 150)

**Fig. 3:**
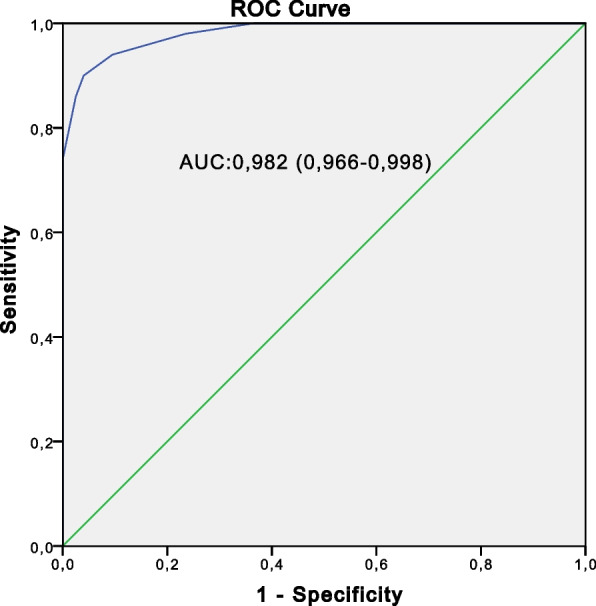
The receiver operating characteristic curve for the global component (C1 + C2) of SpA cases (*n* = 50) versus the healthy control population (*n* = 200)

**Fig. 4:**
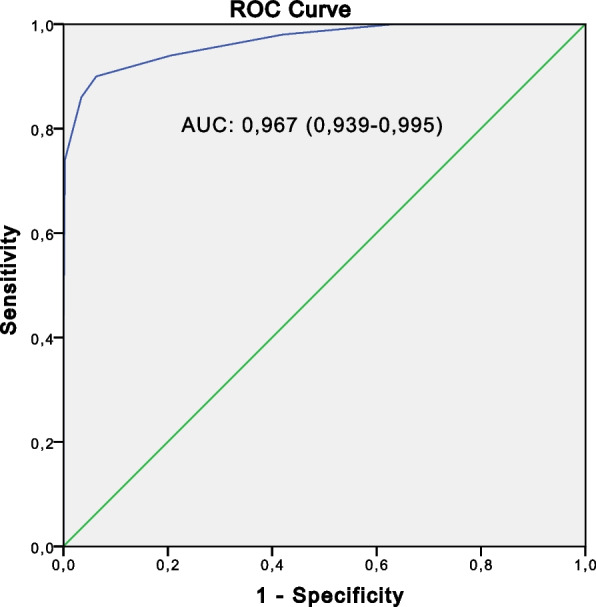
The receiver operating characteristic curve for the global component (C1 + C2) of SpA cases (*n* = 50) versus the aggregated control groups (*n* = 350)

The performance of the global component (C1 + C2) was evaluated using the ROC curve in relation to the two control groups respectively (Figs. 2 and 3) and to the global control groups (Fig. 4) (Table 4).

**Table 4:** Performance of the global component in relation to the different control groups

	Se (%)	Sp (%)	PPV (%)	NPV (%)	AUC
SpA cases vs non-SpA rheumatic controls	97.4	92.0	74.0	99.3	0.947 (0.903–0.991)
SpA cases vs healthy control population	100.0	93.9	74.0	100.0	0.982 (0.966–0.998)
SpA case vs the two control groups	97.4	96.4	74.0	99.7	0.967 (0.939–0.995)

*Se* Sensivity, *Sp* Specificity, *PPV* Positive predictive value, *NPV* Negative predictive value, *AUC* Area under the curve

## Discussion

The prevalence of SpA in Sub-Saharan Africa poorly studied [4, 9] and is low, based on the available studies. Since the mean age of the population in this region is between 30 and 40 years and SpA affects preferentially the young adult population it is therefore important to know the true prevalence. The available classification criteria (including Amor, ESSG and ASAS) cannot be unequivocally applied in Sub-Saharan Africa for several reasons. These criteria were mainly developed in white Caucasian populations and in the western world. Some of the components of these criteria are not well studied in African populations, such as the prevalence of HLA B27 and its relation to SpA. A few studies reported a low prevalence of HLA-B27 antigen [3, 4, 8] possibly reducing the diagnostic information in classification or diagnostic research. Secondly, some elements of the current criteria such as HLA typing and MRI are not widely available and poorly accessible for the entire population. Thirdly, one should note potential problems related to translation into local languages and cultural adaptations of current classification criteria that might be inappropriate for accurate diagnosis or classification. Finally, the clinical spectrum of SpA in Sub-Saharan Africa might be somehow different from the original studies in white Caucasians [19].

Unrestricted application of these existing criteria could likely induce a bias in the prevalence estimate due to reduced sensitivity and specificity. In the present study, we developed and validated a screening tool to identify patients with a high likelihood of having SpA in Kinshasa, the DR Congo. To the best of our knowledge, this was the first study in sub-Saharan Africa based on local data to develop a screening tool for SpA in the population for epidemiological and clinical use. However, a recent US study has developed a screening tool for axial SpA in primary care for general practitioners. Its validity and effectiveness will need to be demonstrated to ensure its widespread application to other populations around the world [20]. The forementioned tool focus only on the detection of axial spondyloarthritis only while the SpASSS questionnaire aims to screen for axial and peripheral spondyloarthritis since peripheral spondyloarthritis is almost equally prevalent in this region [19]. Referral strategies based on context-specific data are necessary to provide accurate case definition and epidemiological data, thus reducing methodological bias. The age at disease onset was relatively older than those described in the Western world [21-24], and the male sex was a stronger discriminator compared to the overall control population. Although the age at the onset of disease was older, the average age of patients with SpA was lower than that of patients with other rheumatic diseases overall. This age at onset of disease was higher in most studies in sub-Saharan Africa and suggests the possibility of a different phenotype [2, 9, 11, 21, 25, 26]. The male sex was preponderant with a sex ratio of 1/0.78, and showed a significant difference comparing with patients suffering from other arthritis. However, this male dominance might be partly explained by clinical importance and gender differences in widespread pain in patients with axial SpA [27-30].

Six items of the screening tool for SpA did not have a significant difference in the response in the 3 groups: “*Are your legs affected?*”, “*Do you have chronic diarrhea?*”, “*Do you have psoriasis?*”, “*Do you have nodule?*”, “*Does one of your family members have AS?*” and “*Does one of your family members have psoriasis/uveitis/chronic diarrhea?*” Indeed, the items with exclusion questions for SpA (“*Are your legs affected?*”, “*Do you have nodules?*”) had similar responses for the 3 groups, giving no statistical difference. On the other hand, the notion of chronic as well as acute diarrhea in our social context can be largely explained by a background of various infectious diseases and non-optimal sanitary conditions, thus this notion of diarrhea was not specifically associated with other items [30].

AS, PsA, and uveitis are not well-known concepts in the population. This could explain the negative responses obtained from the population to these items, although there were some illustrations (photos) to complete understanding of these questions. Two components of the screening tool had obtained a significant convergent and discriminative validity and reliability score of respectively Cronbach's alpha of 0.830 and 0.708. The first component (C1) containing 8 patient-reported question items, achieved an ROC of 0.938, a sensitivity of 92.0% and a specificity of 98.3 comparing with the two control groups (Component 1: *Do you have stiffness in back lasting for* > *30 min? Do you have back pain awakening you the 2* ^*nd*^ *half of the night? Does physical exercise improve your back pain? Does NSAID improve your back pain? Do you have buttock pain? Do you have back pain? Do you have heel pain? Do you have urethritis?*).

The second component included 3 patient-reported question items with an ROC of 0.794, a sensitivity of 86.0 and a specificity of 97.4 comparing the two control groups. (Component 2: *Do you have joint swelling? Do you have joint pain? Do you have joint swelling in more than 3 joints?*). These two components explicitly informed different patterns found in axial SpA and peripheral SpA respectively. The items relating to extra-articular manifestations, notably uveitis and psoriasis, and the notion of family history of SpA did not constitute a considerable association with the other items in the definition of suspect cases. This could be explained by the rarity of extra-articular manifestations found in most of the data reported in sub-Saharan Africa [3, 4, 14, 23, 31, 32].

Urethritis was the only extra-articular manifestation included in the component C1. This is in accordance with the important part of the infection background in our environment explaining the relatively high frequency of reactive arthritis in our context [19]. By grouping together, the items from the two components, our SpASSS questionnaire contained a total of 11 patient-reported question items with 66.9% internal reliability and the number of positive responses expected to referral subject from SpA suspicious cases to confirmation was ≥ 7 positive item responses. Thus, from the 20 items above, only 11 reduction items could be used to define suspicious cases that will have to be confirmed in the second step by a clinical evaluation by the rheumatologist.

The high sensitivity of these two components (92% each), taken together or separately, has shown good performance in the detection of suspicious cases. During the population survey phase, we had a high participation of the population. The low accessibility of rheumatology consultations and the poverty of the population could explain this enthusiasm for attending in the survey. There might therefore be a risk of having a high false positive rate in SpASSS questionnaire. This will require further rigorous evaluation in case definition. A further limitation in this work is that in the SpA group, we indiscriminately took patients without taking into account the duration of their illness and subtypes of SpA. Nevertheless, our results clearly suggest that a set of reported-patient question items can identify a group of patients with a high likelihood of having SpA.

## Conclusion

This validation and item reduction of the SpASSS questionnaire for SpA in Kinshasa, DR Congo might identify patients to refer for case ascertainment. This crucial step is important for conducting reliable and accurate clinical and epidemiological studies in our region in the future.

## Supplementary Information

**Additional file 1. **

**Additional file 2. **

**Additional file 3. **

**Additional file 4. **

**Additional file 5. **

**Additional file 6. **
