# Supplementary material for: Development and validation of a screening tool for SPondyloArthritis Screening in Sub-Saharan Africa: SpASSS questionnaire
Source: BMC Med Res Methodol. 2023 Jun 21;23:145. doi: 10.1186/s12874-023-01966-w (PMC10286346; doi:10.1186/s12874-023-01966-w)
Supplement: Supplementary file 1 — Additional file 1. [file 12874_2023_1966_MOESM1_ESM.docx]

Supplementary file 1.

A systematic literature review was conducted by PLL and JJM. A separate manuscript is prepared about the review process. Methods of the systematic review are briefly presented here. The Preferred Reporting Items for Systematic Reviews and MetaAnalyses (PRISMA) guidelines (19) and the AMSTAR 2 were performed as tools for methodological and reporting characteristics a systematic review. The following online databases were searched: PubMed, Embase and Cochrane addressing questions related to screening questionnaires for referral case of SpA.

*Search strategy*

PubMed, Embase and Cochrane were searched for relevant articles from inception to February 2023.

The search-terms used in both databases can be found in supp table 1.

The reference list of the selected articles was then manually searched for other relevant articles.

Supp Table1

| **Database** | **Search method for SpASSS questionnaire** |
| --- | --- |
| Pubmed | ("Spondylarthritis"[Mesh] OR "Spondylitis, Ankylosing"[Mesh] OR "Spondylarthropathies"[Mesh]) AND ("Surveys and Questionnaires"[Mesh] OR “questionnaires”[Text Word]) |
| Embase | (“ankylosing spondylitis”/exp OR “ankylosing spondylitis” OR “spondyloarthropathy”/exp OR “spondyloarthropathy”) AND (“referral”/exp OR referral) AND (“questionnaire”/exp OR questionnaire) |
| Cochrane | (“spondylitis” OR “spondylarthritides” OR “spondylitis,ankylosing”) AND “Surveys and Questionnaires” |

*Eligibility criteria*

- We included all observational studies on the development and validation of a screening tool for Spondyloarthritis.
- Self-report questionnaires, referral tool or screening questionnaire and protocols concerning
- Studies had to be published in English.
- Only completed and published studies were included.

Supplementary file 1, Figure 1. PRISMA flow chart of our systematic review

Records identified through databases searching

N=4490

PubMed (n=4252); Embase (n=190); Cochrane (n= 48 )

Records after duplicated removed

N=4252

Records screened for eligibility by title/ abstract

N=1058

Full-text articles assessed for eligibility

N=277

Studies included in qualitative synthesis

N=9

Duplicates excluded

N=238

Records excluded after abstract reading

N=3194

- Associated data: 2279
- Books and documents:2
- Meta analysis:60
- Randomized controlled trials: 370
- Review: 933
- Systematic review: 603

Records excluded after full text review

N=781

- N development of questionnaire: 85
- N relevant: 351
- N validation items: 38
- N observational trials:307

Identification

Screening

Eligibility

Included


Supplementary File 1 Table 1 suppl file : Critical appraisal of the included studies AMSTAR 2.

|  | | | | | | | | | | | | | | | | |
| --- | --- | --- | --- | --- | --- | --- | --- | --- | --- | --- | --- | --- | --- | --- | --- | --- |
| Article | Criteria | | | | | | | | | | | | | | | |
|  | 1 | 2 | 3 | 4 | 5 | 6 | 7 | 8 | 9 | 10 | 11 | 12 | 13 | 14 | 15 | 16 |
| Shridharmurthy D et al. (2022) | Y | N | Y | Y | Y | Y | Y | Y | Y | Y | NA | NA | NA | NA | NA | Y |
| Shridharmurthy D et al. (2022 | Y | N | Y | Y | Y | N | N | Y | Y | Y | NA | NA | NA | NA | NA | Y |
| Phillips R et al. (2022) | Y | Y | N | Y | Y | Y | Y | N | N | Y | NA | NA | NA | NA | NA | Y |
| Lapane KL et al. (2021) | Y | N | N | Y | Y | N | Y | Y | Y | Y | NA | NA | NA | NA | NA | N |
| Variola A et al.al. (2020) | Y | N | N | Y | Y | N | Y | Y | N | Y | NA | NA | NA | NA | NA | Y |
| Xiang L et al. (2021) | Y | Y | N | Y | Y | Y | Y | Y | Y | Y | NA | NA | NA | NA | NA | Y |
| Kwan YH et al. (2018) | Y | Y | N | Y | Y | Y | Y | Y | Y | Y | NA | NA | NA | NA | NA | Y |
| Hamilton L et al. (2013) | Y | Y | N | Y | Y | Y | N | Y | Y | Y | NA | NA | NA | NA | NA | N |
| Weisman MH et al. (2010) | Y | N | Y | N | Y | N | Y | N | N | Y | NA | NA | NA | NA | NA | Y |
