# Supplementary material for: Development and validation of a screening tool for SPondyloArthritis Screening in Sub-Saharan Africa: SpASSS questionnaire
Source: BMC Med Res Methodol. 2023 Jun 21;23:145. doi: 10.1186/s12874-023-01966-w (PMC10286346; doi:10.1186/s12874-023-01966-w)
Supplement: Supplementary file 2 — Additional file 2. [file 12874_2023_1966_MOESM2_ESM.docx]

Supplementary file 2.

SpASSS questionnaire (C1 + C2)

|  | YES | NO |
| --- | --- | --- |
| 1. *Do you have stiffness in back lasting for >30 minutes?* |  |  |
| 1. *Do you have back pain awakening you the 2^nd^ half of the night?* |  |  |
| 1. *Does physical exercise improve your back pain?* |  |  |
| 1. *Does NSAID improve your back pain?* |  |  |
| 1. *Do you have buttock pain?* |  |  |
| 1. *Do you have back pain?* |  |  |
| 1. *Do you have heel pain?* |  |  |
| 1. *Do you have urethritis ?* |  |  |
| 1. *Do you have joint swelling?* |  |  |
| 1. *Do you have joint pain?* |  |  |
| 1. *Do you have joint swelling in more than 3 joints?* |  |  |

**Any case of suspected SpA to be referred was defined by a score of at least 7/11 positive questions**

(Sensitivity: 97.4 %, Specificity 96.4%, positive predictive value 74.0%, negative predictive value 99.7%, area under the curve: 0.967)
