# Supplementary material for: Development and validation of a screening tool for SPondyloArthritis Screening in Sub-Saharan Africa: SpASSS questionnaire
Source: BMC Med Res Methodol. 2023 Jun 21;23:145. doi: 10.1186/s12874-023-01966-w (PMC10286346; doi:10.1186/s12874-023-01966-w)
Supplement: Supplementary file 3 — Additional file 3. [file 12874_2023_1966_MOESM3_ESM.docx]

**Supplements**

Supplementary table 1. Extraction coefficient after analysis of variance of different items of the screening questionnaire

| N° | Items | Extraction Coefficient |
| --- | --- | --- |
| 1 | Do you have joint pain? | 0.679 |
| 2 | Do you have joint swelling? | 0.831 |
| 3 | Do you have joint swelling in more than 3 joints? | 0.692 |
| 4 | Are your legs affected ? | 0.666 |
| 5 | Do you have back pain? | 0.634 |
| 6 | Do you have stiffness in back lasting for >30’? | 0.769 |
| 7 | Do you have back pain a wakening you the 2^nd^ half of the night? | 0.701 |
| 8 | Does physical exercise improve your back pain? | 0.621 |
| 9 | Does NSAID improve your back pain? | 0.680 |
| 10 | Do you have anterior chest pain? | 0.821 |
| 11 | Do you have buttock pain? | 0.605 |
| 12 | Do you have red eyes now/in past? | 0.582 |
| 13 | Do you have heel pain? | 0.359 |
| 14 | Do you have chronic diarrhea? | 0.644 |
| 15 | Do you have urethritis ? | 0.416 |
| 16 | Do you have psoriasis ? | 0.735 |
| 17 | Does one of your family members has AS? | 0.784 |
| 18 | Does one of your family members has psoriasis/ uveitis/chronic diarrhea? | 0.541 |
| 19 | Do you have nodules? | 0.743 |
| 20 | Do you have dactylitis? | 0.680 |
