# Supplementary material for: Development and validation of a screening tool for SPondyloArthritis Screening in Sub-Saharan Africa: SpASSS questionnaire
Source: BMC Med Res Methodol. 2023 Jun 21;23:145. doi: 10.1186/s12874-023-01966-w (PMC10286346; doi:10.1186/s12874-023-01966-w)

| **C1: the 1^st^ component**  *Do you have stiffness in back lasting for >30 minutes ?*  *Do you have back pain awakening you the 2^nd^ half of the night ?*  *Does physical exercise improve your back pain ?*  *Does NSAID improve your back pain ?*  *Do you have buttock pain ?*  *Do you have back pain ?*  *Do you have heel pain ?*  *Do you have urethritis ?* | **C2 : the 2^nd^ component**  *Do you have joint swelling ?*  *Do you have joint pain ?*  *Do you have joint swelling in more than 3 joints ?* |
| --- | --- |

Supplementary figure 2. Performance of the two component items of screening tool between the SpA cases and the healthy control population (n= 200)


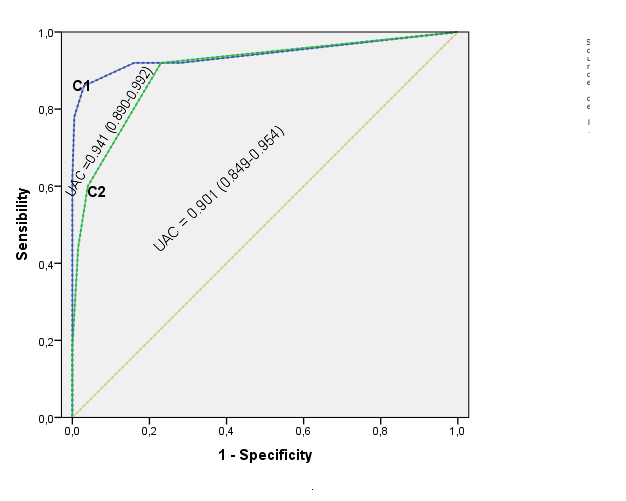

Supplement: Supplementary file 5 — Additional file 5. [file 12874_2023_1966_MOESM5_ESM.docx]
