# Supplementary material for: Development and validation of a screening tool for SPondyloArthritis Screening in Sub-Saharan Africa: SpASSS questionnaire
Source: BMC Med Res Methodol. 2023 Jun 21;23:145. doi: 10.1186/s12874-023-01966-w (PMC10286346; doi:10.1186/s12874-023-01966-w)
Supplement: Supplementary file 6 — Additional file 6. [file 12874_2023_1966_MOESM6_ESM.docx]

Supplementary figure 3 . The receiver operating characteristic curves for the 2 components (C1+C2) of the SpA cases versus to the aggregated control groups(n=350)


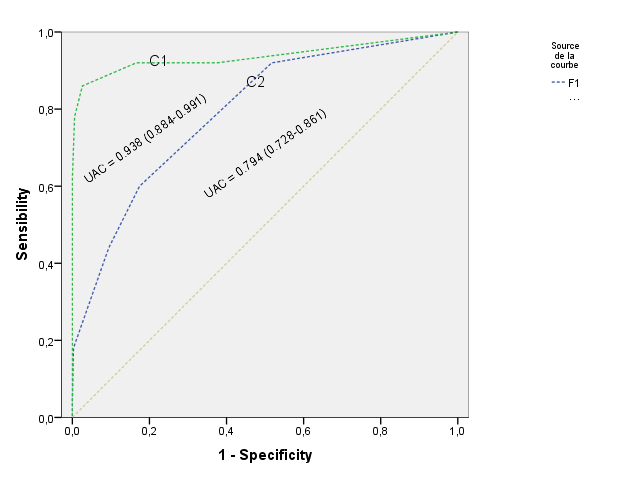


| **C1: first component**  *Do you have stiffness in back lasting for >30 minutes ?*  *Do you have back pain awakening you the 2^nd^ half of the night ?*  *Does physical exercise improve your back pain ?*  *Does NSAID improve your back pain ?*  *Do you have buttock pain ?*  *Do you have back pain ?*  *Do you have heel pain ?*  *Do you have urethritis ?* | **C2 : second component**  *Do you have joint swelling ?*  *Do you have joint pain ?*  *Do you have joint swelling in more than 3 joints ?* |
| --- | --- |
